# Genetic polymorphisms in *CYP4F2* may be associated with lung cancer risk among females and no-smoking Chinese population

**DOI:** 10.3389/fonc.2023.1114218

**Published:** 2023-03-14

**Authors:** Hongyang Shi, Yonghong Zhang, Yu Wang, Ping Fang, Yun Liu

**Affiliations:** Department of Respiratory and Critical Care Medicine, the Second Affiliated Hospital of Xi’an Jiaotong University, Xi’an, China

**Keywords:** *CYP4F2*, lung cancer, single nucleotide polymorphisms, smoking, gender

## Abstract

**Background:**

Our study aimed to explore the potential association of *CYP4F2* gene polymorphisms with lung cancer (LC) risk.

**Methods:**

The five variants in *CYP4F2* were genotyped using Agena MassARRAY in 507 cases and 505 controls. Genetic models and haplotypes based on logistic regression analysis were used to evaluate the potential association between *CYP4F2* polymorphisms and LC susceptibility.

**Results:**

This study observed that rs12459936 was linked to an increased risk of LC in no-smoking participants (allele: OR = 1.38, *p* = 0.035; homozygote: OR = 2.00, *p* = 0.035; additive: OR = 1.40, *p* = 0.034) and females (allele: OR = 1.64, *p* = 0.002; homozygote: OR = 2.57, *p* = 0.006; heterozygous: OR = 2.56, *p* = 0.001; dominant: OR = 2.56, *p <* 0.002; additive: OR = 1.67, *p* = 0.002). Adversely, there was a significantly decreased LC risk for rs3093110 in no-smoking participants (heterozygous: OR = 0.56, *p* = 0.027; dominant: OR = 0.58, *p* = 0.035), rs3093193 (allele: OR = 0.66, *p* = 0.016; homozygote: OR = 0.33, *p* = 0.011; recessive: OR = 0.38, *p* = 0.021; additive: OR = 0.64, *p* = 0.014), rs3093144 (recessive: OR = 0.20, *p* = 0.045), and rs3093110 (allele: OR = 0.54, *p* = 0.010; heterozygous: OR = 0.50, *p* = 0.014; dominant: OR = 0.49, *p* = 0.010; additive: OR = 0.54, *p* = 0.011) in females.

**Conclusions:**

The study demonstrated that *CYP4F2* variants were associated with LC susceptibility, with evidence suggesting that this connection may be affected by gender and smoking status.

## Introduction

Lung cancer (LC) has been regarded as one of the most common causes of cancer-related death worldwide over the past few decades, with an estimated 2.1 million new diagnoses of LC in 2018, accounting for 12% of the total increase in cancer cases (1). In recent years, the incidence of LC in China has been consistent with the global trend, showing a rapid increase, and LC has since become the main cause of cancer-related deaths in China (2). It is predicted that the mortality of LC in China is likely to increase by about 40% between 2015 and 2030 (3). Despite advances in early detection, the majority of LC patients are often diagnosed at a later stage, resulting in a 5-year overall survival rate of only 10% to 15%, according to statistics (4). The burden of LC on our society is increasing day by day and cannot be ignored. Various factors can predispose people to LC, with smoking being the most prevalent factor. In addition, other potential risk factors include gender, age, race, ethnicity, and especially single nucleotide polymorphisms (SNPs) (5, 6).

Cytochrome P450s (CYP), phase I drug metabolizing enzymes, encode 57 CYP proteins in the human genome and are responsible for the metabolism of numerous endogenous and xenobiotic compounds (7). The *CYP4F2* gene, a member of the *CYP450* superfamily, is an ω-hydroxylase that catalyzes the first step of the vitamin E metabolic pathway (8), as well as the metabolism of arachidonic acid (AA) to generate 20-hydroxyethyl hexadecanoic acid (20-HETE) through ω-hydroxylation (9). 20-HETE is known to promote tumorigenesis by increasing a variety of pro-inflammatory mediators, cytokines, and chemokines. Previous studies have demonstrated that the elevated expression of *CYP4F2* enzymes and 20-HETE is closely related to ovarian cancer (10). We hypothesized that *CYP4F2* might be involved in tumor genesis and development by accelerating the production of 20-HETE. Additionally, Geng et al. have proved that rs1558139 and rs2108622 of *CYP4F2* are associated with hypertension, and the association between rs1558139 and hypertension is particularly strong in men (11). Despite this, there is a lack of studies investigating the association between *CYP4F2* polymorphisms and LC risk.

In this case–control study, five SNPs (rs3093203, rs3093144, rs12459936, rs3093110, and rs3093193) in *CYP4F2* were genotyped by the Agena MassARRAY platform. The gender- and smoking-stratified analyses on the correlation between *CYP4F2* variants and LC risk were performed.

## Materials and methods

### Study subjects

A total of 507 newly diagnosed LC patients (353 males and 154 females) were randomly recruited from the Second Affiliated Hospital of Xi’an Jiaotong University in the case–control association analysis between *CYP4F2* polymorphisms and the risk of LC. All patients had no history of any other cancers and had not received chemotherapy before acquiring blood samples. Further, the control group comprised 505 unrelated healthy controls (354 males and 151 females) from the physical examination center of the hospital. Information about all subjects, including age, gender, height (cm), weight (kg), smoking status, drinking status, tumor stage, and lymph node metastasis, was collected from questionnaires and clinical data. Peripheral blood samples were collected from all study subjects into vacutainer tubes containing EDTA, and genomic DNA was then isolated from the collected blood samples using the GoldMag-Mini Purification Kit (GoldMag Co. Ltd., Xi’an, China) and stored at −80°C. DNA concentration and purity were determined by a NanoDrop 2000 spectrophotometer (Thermo Fisher Scientific, Waltham, MA, USA).

### SNP selection and genotyping

In this study, five SNPs (rs3093203, rs3093144, rs12459936, rs3093110, and rs3093193) in *CYP4F2* were selected according to previously published studies on the association between *CYP4F2* polymorphisms and disease susceptibility (12–14). The genotype distributions of the candidate SNPs in controls met Hardy–Weinberg equilibrium (HWE) (*p >*0.05). All the candidate SNPs had a minor allele frequency (MAF) of >5% in the Han Chinese in Beijing (CHB) population from the 1,000 Genomes Project (http://www.internationalgenome.org/). The primers for five SNPs were designed by Agena Bioscience Assay Design Suite version 2.0 software. The polymorphisms were genotyped using the Agena MassARRAY platform (Agena Bioscience, San Diego, CA, USA) with iPLEX gold chemistry. Ultimately, Agena Bioscience TYPER version 4.0 software was used for data management and genotyping result analysis.

### Expression analysis

We extracted the data for *CYP4F2* expression in normal lung tissues and lung squamous cell carcinoma (LUSC) tissues under different subgroups from the TCGA database and analyzed them *via* UALCAN (http://ualcan.path.uab.edu/index.html), which is an interactive web resource for tumor subgroup gene expression analysis and survival analysis.

### Statistical analysis

SPASS version 22.0 software was applied for statistical analysis. HWE was calculated for the control group by the chi-square test. Differences in the continuous characteristic (age) and categorical variable (gender) between patients with LC and controls were measured by the student’s t-test and Pearson Chi-Square test, respectively. The correlation between *CYP4F2* variants and LC susceptibility was evaluated by logistic regression analysis adjusted for age and gender using PLINK software (version 1.07) under multiple genetic models (allele, genotype, dominant, recessive, and additive). Odds ratio (OR) and 95% confidence interval (CI) were calculated to assess the relationship between *CYP4F2* SNPs and LC risk (OR = 1: no impact; OR <1: protective factor; OR >1: risk factor). Finally, PLINK (version 1.07) and Haploview (version 4.2) softwares were used to analyze the pairwise linkage disequilibrium (LD) among five SNPs and generate an LD map to observe the linkage degree among them based on D’ and r-squared values. The SNPStats software (https://www.snpstats.net/start.htm) was used to estimate the correlation between *CYP4F2* haplotypes and LC risk. In our study, the *p*-values of all tests were two-sided, and *p <*0.05 was considered statistically significant.

## Results

### Participant characteristics

The mean ages of 507 LC patients and 505 unrelated healthy controls were 61.30 ± 8.32 years and 58.91 ± 9.58 years, respectively (**Table 1**). In our study, there were no statistically significant differences in age (*p* = 0.525) and gender (*p* = 0.870) distribution between cases and controls.

**Table 1:** Characteristics of patients with lung cancer and controls.

Variables	Cases (N = 507)	Controls (N = 505)	*p-*value
Age (mean ± SD), years	61.30 ± 8.32	58.91 ± 9.58	0.525
>60	271 (53%)	270 (53%)	0.973
≤60	236 (47%)	235 (47%)	
Sex			0.870
Male	353 (70%)	354 (70%)	
Female	154 (30%)	151 (30%)	
BMI (kg/m^2^)			
<24	316 (62%)	146 (29%)	
≥24	177 (35%)	161 (32%)	
Absence	14 (3%)	198 (39%)	
Smoking status			
Yes	251 (50%)	136 (27%)	
No	250 (49%)	140 (28%)	
Absence	6 (1%)	229 (45%)	
Drinking status			
Yes	114 (22%)	109 (22%)	
No	356 (70%)	135 (27%)	
Absence	27 (8%)	261 (51%)	
Histology			
Adenocarcinoma	187 (37%)		
Squamous	119 (23%)		
Absence	201 (40%)		
LN metastasis			
Yes	214 (42%)		
No	84 (17%)		
Absence	209 (41%)		
Stage			
I, II	83 (16%)		
III, IV	260 (51%)		
Absence	164 (33%)		

BMI, body mass index; LN, lymph node.

p <0.05 indicates statistical significance.

### Basic information about the selected SNPs in *CYP4F2*

The basic information about the five SNPs in *CYP4F2* (rs3093203, rs3093144, rs12459936, rs3093110, and rs3093193) among cases and controls was displayed (**Table 2**), including gene, SNP ID, position, alleles, HWE, and OR (95% CI). The five SNPs in controls were in accordance with HWE (*p >*0.05). We further evaluated the association between the five SNPs and LC susceptibility by logistic regression (**Table 2**). The four genetic models (genotype, dominant, recessive, and additive) were also applied to analyze the association by logistic regression adjusted for age and gender (**Table S1**). Unfortunately, there was no significant association between these five SNPs in *CYP4F2* and LC susceptibility under the allelic and genetic models.

**Table 2:** Basic information and allele frequencies of candidate SNPs in *CYP4F2*.

SNP ID	Position	Alleles	Role	MAF		HWE *p*-value	OR (95% CI)	*p*-value
		A/B		Case	Control			
rs3093203	Chr19:15878374	C/T	3’UTR	0.240	0.229	1.000	1.06 (0.86–1.31)	0.558
rs3093193	Chr19:15881104	C/G	intronic	0.288	0.301	0.525	0.94 (0.78–1.14)	0.533
rs12459936	Chr19:15882231	C/T	intronic	0.463	0.450	0.720	1.05 (0.88–1.26)	0.557
rs3093144	Chr19:15891487	A/G	intronic	0.187	0.172	0.755	1.11 (0.88–1.39)	0.377
rs3093110	Chr19:15896974	C/T	intronic	0.105	0.129	0.694	0.79 (0.06–1.03)	0.084

SNP, single-nucleotide polymorphism; Chr, chromosome; MAF, minor allele frequency; HWE, Hardy–Weinberg equilibrium; OR, odds ratio; 95% CI, 95% confidence interval.

A/B: minor/major allele in the controls; ORs (95% CI) were calculated by logistic regression; p-values were calculated by Pearson χ^2^ test.

### Stratification analysis by smoking status

The smoking-stratified analysis (**Table 3**) was performed to examine the relationship between *CYP4F2* variants and LC risk. Our results showed that rs12459936 in *CYP4F2* was associated with an increased risk of LC in no-smoking individuals under the allele (T *vs*. C: OR = 1.38, 95% CI: 1.02–1.85, *p* = 0.035), genotype (TT *vs*. CC: OR = 2.00, 95% CI: 1.05–3.82, *p* = 0.035), and additive (OR = 1.40, 95% CI: 1.03–1.92, *p* = 0.034) models. On the contrary, rs3093110 was found to have a protective effect against LC risk in no-smoking individuals under the genotype (GA *vs*. AA: OR = 0.56, 95% CI: 0.33–0.94, *p* = 0.027) and dominant (GG + GA *vs*. AA: OR = 0.58, 95% CI: 0.35–0.96, *p* = 0.035) models.

**Table 3:** The association of variants in *CYP4F2* with lung cancer susceptibility stratified by smoking status.

SNP ID	Models	Genotypes	No smoking				Smoking			
			Cases (%)	Controls (%)	OR (95% CI)	*p*-value	Cases (%)	Controls (%)	OR (95% CI)	*p*-value
rs12459936	Allele	C	259 (51.8%)	167 (59.6%)	1		279 (55.6%)	151 (55.5%)	1	
		T	241 (48.2%)	113 (40.4%)	1.38 (1.02–1.85)	**0.035**	223 (44.4%)	121 (44.5%)	1.00 (0.74–1.34)	0.987
	Genotype	CC	63 (25.2%)	47 (33.6%)	1		83 (33.1%)	45 (33.1%)	1	
		TT	54 (21.6%)	20 (14.3%)	2.00 (1.05–3.82)	**0.035**	55 (21.9%)	30 (22.1%)	1.00 (0.56–1.77)	0.993
		TC	133 (53.2%)	73 (52.1%)	1.35 (0.83–2.18)	0.223	113 (45.0%)	61 (44.9%)	1.00 (0.62–1.62)	0.989
	Dominant	CC	63 (25.2%)	47 (33.6%)	1		83 (33.1%)	45 (33.1%)	1	
		TT + TC	187 (74.8%)	93 (66.4%)	1.49 (0.94–2.35)	0.090	168 (66.9%)	91 (66.9%)	1.00 (0.64–1.56)	0.995
	Recessive	TC + CC	196 (78.4%)	120 (85.7%)	1		196 (78.1%)	106 (77.9%)	1	
		TT	54 (21.6%)	20 (14.3%)	1.65 (0.94–2.91)	0.084	55 (21.9%)	30 (22.1%)	1.00 (0.60–1.65)	0.986
	Additive	TT + TC + CC	–	–	1.40 (1.03–1.92)	**0.034**	–	–	1.00 (0.75–1.33)	0.996
rs3093110	Allele	A	453 (90.6%)	243 (86.8%)	1		445 (88.6%)	244 (90.4%)	1	
		G	47 (9.4%)	37 (13.2%)	0.68 (0.43–1.08)	0.099	57 (11.4%)	26 (9.6%)	1.20 (0.74–1.96)	0.461
	Genotype	AA	206 (82.4%)	104 (74.3%)	1		198 (78.9%)	111 (82.2%)	1	0.719
		GG	3 (1.2%)	1 (0.7%)	1.26 (0.13–12.41)	0.844	4 (1.6%)	2 (1.5%)	1.09 (0.20–6.04)	0.923
		GA	41 (16.4%)	35 (25.0%)	0.56 (0.33–0.94)	**0.027**	49 (19.5%)	22 (16.3%)	1.26 (0.72–2.20)	0.417
	Dominant	AA	206 (82.4%)	104 (74.3%)	1		198 (78.9%)	111 (82.2%)	1	
		GG + GA	44 (17.6%)	36 (25.7%)	0.58 (0.35–0.96)	**0.035**	53 (21.1%)	24 (17.8%)	1.25 (0.73–2.13)	0.425
	Recessive	GA + AA	247 (98.8%)	139 (99.3%)	1		247 (98.4%)	133 (98.5%)	1	
		GG	3 (1.2%)	1 (0.7%)	1.44 (0.15–14.16)	0.755	4 (1.6%)	2 (1.5%)	1.04 (0.19–5.78)	0.961
	Additive	GG + GA + AA	–	–	0.63 (0.40–1.02)	0.590	–	–	1.20 (0.74–1.94)	0.468

SNP, single-nucleotide polymorphism; BMI, body mass index; OR, odds ratio; 95% CI, 95% confidence interval.

Bold values are statistically significant; OR (95% CI) and p-values were computed by logistic regression analysis with adjustments for age and gender.

### Stratification analysis by gender

In addition, the analysis stratified by gender (**Table 4**) demonstrated that rs3093193 (G *vs*. C: OR = 0.66, 95% CI: 0.47–0.92, *p* = 0.016; GG *vs*. CC: OR = 0.33, 95% CI: 0.14–0.77, *p* = 0.011; GG *vs*. GC + CC: OR = 0.38, 95% CI: 0.17–0.86, *p* = 0.021; additive: OR = 0.64, 95% CI: 0.45–0.91, *p* = 0.014) was related to a decreased risk of LC in females. Rs3093144 in the recessive model (TT *vs*. TC + CC: OR = 0.20, 95% CI: 0.04–0.96, *p* = 0.045) and rs3093110 in the allele, genotype, dominant, and additive models (G *vs*. A: OR = 0.54, 95% CI: 0.33–0.87, *p* = 0.010; GA *vs*. AA: OR = 0.50, 95% CI: 0.29–0.87, *p* = 0.014; GG + GA *vs*. AA: OR = 0.49, 95% CI: 0.29–0.84, *p* = 0.010; additive: OR = 0.54, 95% CI: 0.33–0.87, *p* = 0.011) showed a protective effect on LC in females. However, the *CYP4F2* rs12459936 was associated with an increased risk of LC in females under the allele, genotype, dominant, and additive models (T *vs*. C: OR = 1.64, 95% CI: 1.19–2.27, *p* = 0.002; TT *vs*.CC: OR = 2.57, 95% CI: 1.49–4.39, *p* = 0.006; TC *vs*.CC: OR = 2.56, 95% CI: 1.49–4.39, *p* = 0.001; TT + TC *vs*.CC: OR = 2.56, 95% CI: 1.53–4.28, *p <*0.001; additive: OR = 1.67, 95% CI: 1.20–2.33, *p* = 0.002).

**Table 4:** The association of variants in *CYP4F2* with lung cancer susceptibility stratified by gender.

SNP ID	Models	Genotypes	Males				Females			
			Cases (%)	Controls (%)	OR (95% CI)	*p*-value	Cases (%)	Controls (%)	OR (95% CI)	*p*-value
rs3093193	Allele	C	500 (70.8%)	515 (72.9%)	1		222 (72.1%)	190 (62.9%)	1	
		G	206 (29.2%)	191 (27.1%)	1.11 (0.88–1.40)	0.375	86 (27.9%)	112 (37.1%)	0.66 (0.47–0.92)	**0.016**
	Genotype	CC	179 (50.7%)	183 (51.8%)	1		77 (50.0%)	60 (39.7%)	1	
		GG	32 (9.1%)	21 (5.9%)	1.59 (0.88–2.87)	0.124	9 (5.8%)	21 (13.9%)	0.33 (0.14–0.77)	**0.011**
		GC	142 (40.2%)	149 (42.2%)	0.98 (0.72–1.34)	0.911	68 (44.2%)	70 (46.4%)	0.75 (0.47–1.21)	0.243
	Dominant	CC	179 (50.7%)	183 (51.8%)	1		77 (50.0%)	60 (39.7%)	1	
		GG + GC	174 (49.3%)	170 (48.2%)	1.06 (0.79–1.42)	0.717	77 (50.0%)	91 (60.3%)	0.66 (0.42–1.04)	0.071
	Recessive	GC + CC	321 (90.9%)	332 (94.1%)	1		145 (94.2%)	130 (86.1%)	1	
		GG	32 (9.1%)	21 (5.9%)	1.60 (0.90–2.84)	0.107	9 (5.8%)	21 (13.9%)	0.38 (0.17–0.86)	**0.021**
	Additive	GG + GC + CC	–	–	1.12 (0.89–1.42)	0.333	–	–	0.64 (0.45–0.91)	**0.014**
rs12459936	Allele	C	395 (55.9%)	372 (52.5%)	1		150 (48.7%)	184 (60.9%)	1	
		T	311 (44.1%)	336 (47.5%)	0.87 (0.71–1.08)	0.199	158 (51.3%)	118 (39.1%)	1.64 (1.19–2.27)	**0.002**
	Genotype	CC	116 (32.9%)	95 (26.8%)	1		32 (20.8%)	60 (39.7%)	1	
		TT	74 (21.0%)	77 (21.8%)	0.79 (0.52–1.00)	0.269	36 (23.4%)	27 (17.9%)	2.57 (1.32–5.00)	**0.006**
		TC	163 (46.2%)	182 (51.4%)	0.74 (0.52–1.05)	0.088	86 (55.8%)	64 (42.4%)	2.56 (1.49–4.39)	**0.001**
	Dominant	CC	116 (32.9%)	95 (26.8%)	1		32 (20.8%)	60 (39.7%)	1	
		TT + TC	237 (67.1%)	259 (73.2%)	0.76 (0.55–1.05)	0.090	122 (79.2%)	91 (60.3%)	2.56 (1.53–4.28)	**<0.001**
	Recessive	TC + CC	279 (79.0%)	277 (78.2%)	1		118 (76.6%)	130 (82.1%)	1	
		TT	74 (21.0%)	77 (21.8%)	0.95 (0.66–1.36)	0.784	36 (23.4%)	27 (17.9%)	1.41 (0.80–2.47)	0.233
	Additive	TT + TC + CC	–	–	0.88 (0.71–1.08)	0.212	–	–	1.67 (1.20–2.33)	**0.002**
rs3093144	Allele	C	571 (80.9%)	595 (84%)	1		253 (82.1%)	241 (79.8%)	1	
		T	135 (19.1%)	113 (16%)	1.25 (0.95–1.64)	0.118	55 (17.9%)	61 (20.2%)	0.86 (0.57–1.29)	0.461
	Genotype	CC	232 (65.7%)	248 (70.1%)	1		101 (65.6%)	99 (65.6%)	1	
		TT	14 (4.0%)	7 (2.0%)	2.15 (0.85–5.43)	0.105	2 (1.3%)	9 (6.0%)	0.21 (0.04–1.02)	0.053
		TC	107 (30.3%)	99 (28.0%)	1.16 (0.84–1.61)	0.370	51 (33.1%)	43 (28.5%)	1.16 (0.71–1.90)	0.549
	Dominant	CC	232 (65.7%)	248 (70.1%)	1		101 (65.6%)	99 (65.6%)	1	
		TT + TC	121 (34.3%)	106 (29.9%)	1.23 (0.89–1.68)	0.206	53 (34.4%)	52 (34.4%)	1.00 (0.62–1.60)	0.997
	Recessive	TC + CC	339 (96.0%)	347 (98.0%)	1		152 (98.7%)	142 (94.0%)	1	
		TT	14 (4.0%)	7 (2.0%)	2.06 (0.82–5.16)	0.125	2 (1.3%)	9 (6.0%)	0.20 (0.04–0.96)	**0.045**
	Additive	TT + TC + CC	–	–	1.25 (0.95–1.65)	0.110	–	–	0.86 (0.57–1.29)	0.460
rs3093110	Allele	A	631 (89.4%)	626 (88.9%)	1		277 (89.9%)	250 (82.8%)	1	
		G	75 (10.6%)	78 (11.1%)	0.95 (0.68–1.33)	0.783	31 (10.1%)	52 (17.2%)	0.54 (0.33–0.87)	**0.010**
	Genotype	AA	283 (80.2%)	277 (78.7%)	1		125 (81.2%)	103 (68.2%)	1	
		GG	5 (1.4%)	3 (0.9%)	1.70 (0.40–7.19)	0.474	2 (1.3%)	4 (2.6%)	0.42 (0.07–2.32)	0.318
		GA	65 (18.4%)	72 (20.5%)	0.89 (0.61–1.30)	0.549	27 (17.5%)	44 (29.1%)	0.50 (0.29–0.87)	**0.014**
	Dominant	AA	283 (80.2%)	277 (78.7%)	1		125 (81.2%)	103 (68.2%)	1	
		GG + GA	70 (19.8%)	75 (21.3%)	0.92 (0.64–1.33)	0.668	29 (18.8%)	48 (31.8%)	0.49 (0.29–0.84)	**0.010**
	Recessive	GA + AA	348 (98.6%)	349 (99.1%)	1		152 (98.7%)	147 (97.4%)	1	
		GG	5 (1.4%)	3 (0.9%)	1.74 (0.41–7.35)	0.454	2 (1.3%)	4 (2.6%)	0.48 (0.09–2.68)	0.405
	Additive	GG + GA + AA	–	–	0.96 (0.69–1.35)	0.832	–	–	0.54 (0.33–0.87)	**0.011**

Bold values are statistically significant; p-values were computed by logistic regression analysis with adjustment for age.

SNP, single-nucleotide polymorphism; BMI, body mass index; OR, odds ratio; 95% CI, 95% confidence interval.

### Haplotype analysis

**Figure 1:**
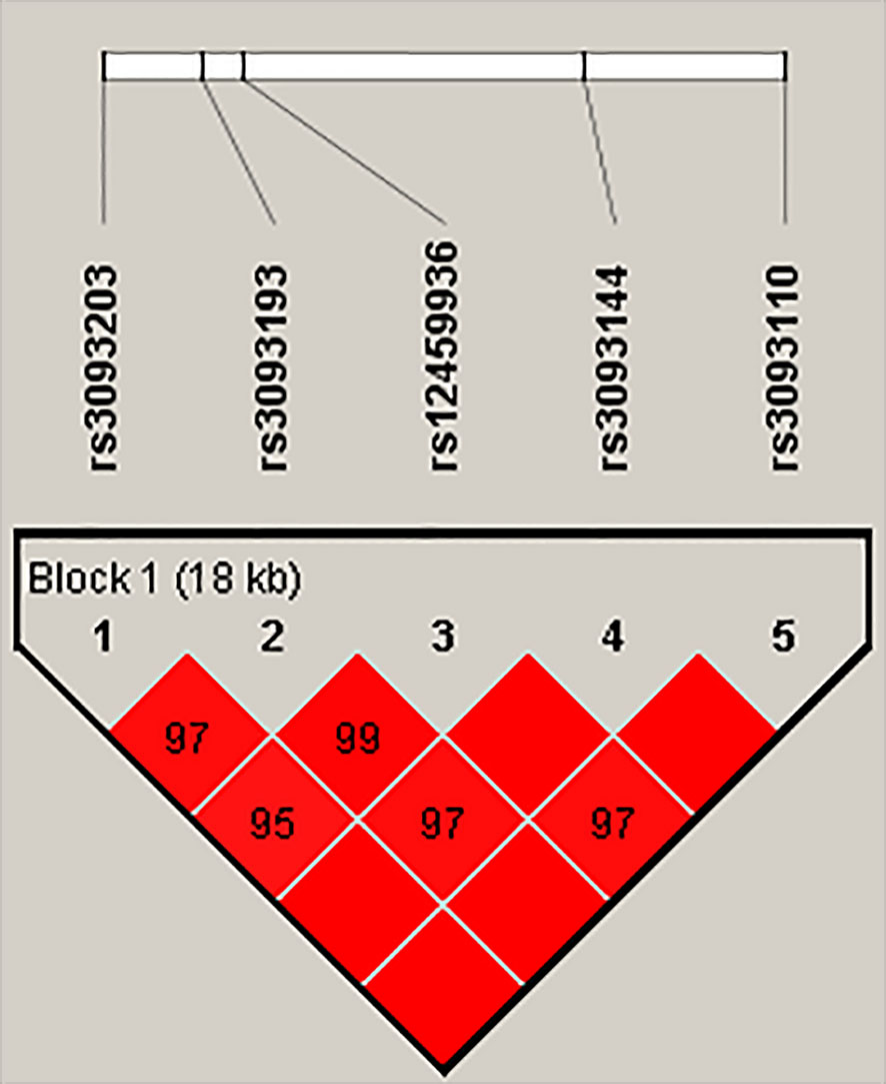
Haplotype block map for SNPs in the *CYP4F2* gene. The numbers inside the diamonds indicate the D′ value × 100 for pairwise analyses.

Finally, the results of haplotype analysis indicated a strong 18-kb LD block among the five SNPs (rs3093203, rs3093193, rs12459936, rs3093144, and rs3093110) (**Figure 1** and **Table S2**). Compared with haplotype “GCTCA,” haplotypes “GGCTA” (OR = 0.63, 95% CI: 0.40–1.00, *p* = 0.048) and “GGCCG” (OR = 0.46, 95% CI: 0.27–0.78, *p* = 0.004) were associated with a decreased risk of LC in females (**Table 5**). For non-smokers, the haplotype “GGCCG” (OR = 0.54, 95% CI: 0.32–0.90, *p* = 0.046) was also associated with decreased susceptibility to LC (**Table 5**).

**Table 5:** The frequency of *CYP4F2* haplotypes and their association with the risk of lung cancer in subgroups.

SNP ID	Haplotypes	Female				No-smoker			
		Controls-Fre	Cases-Fre	OR (95% CI)	*p*-value	Controls-Fre	Cases-Fre	OR (95% CI)	*p*-value
rs3093203|rs3093193|rs12459936|rs3093144|rs3093110	GCTCA	0.387	0.509	1		0.404	0.479	1	
	ACCCA	0.211	0.184	0.64 (0.40–1.02)	0.060	0.246	0.219	0.79 (0.53–1.17)	0.250
	GGCTA	0.199	0.166	0.63 (0.40–1.00)	**0.048**	0.182	0.168	0.72 (0.47–1.12)	0.140
	GGCCG	0.162	0.101	0.46 (0.27–0.78)	**0.004**	0.132	0.092	0.54 (0.32–0.90)	**0.019**
	GCCCA	0.031	0.020	0.50 (0.17–1.52)	0.220	0.029	0.021	0.57 (0.21–1.56)	0.280

SNP, single nucleotide polymorphism, OR, odds ratio, CI, confidence interval.

p <0.05 indicates statistical significance. Significant values are marked in bold.

### Bioinformatics analysis of *CYP4F2* expression in LC

**Figure 2:**
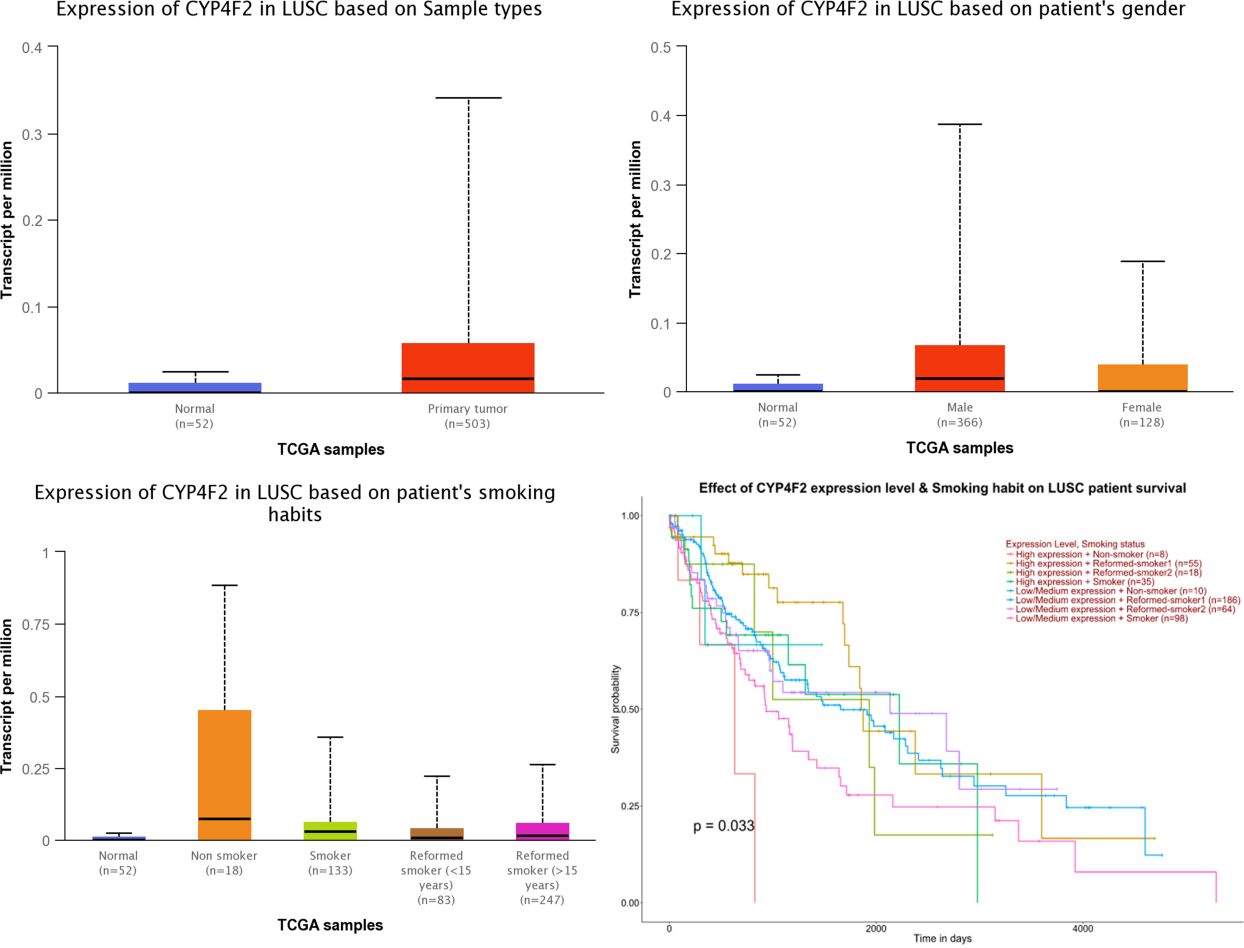
The expression of *CYP4F2* in normal lung squamous cell carcinoma tissues based on different types.

The analysis of the expression level of *CYF4F2* in normal and LUSC tissues and its effect on the survival of these patients was conducted using UALCAN online analysis software based on the TCGA database, as shown in **Figure 2**. We observed that the expression level of *CYP4F2* was significantly different between normal and LUSC tissues (*p <*0.001). In addition, the expression level of *CYP4F2* was higher in non-smoking LUSC patients than in normal and smoking ones (*p <*0.001). The expression level was higher in males than in females (*p <*0.001). Moreover, a high expression level of *CYP4F2* was found to be significantly related to the poor prognosis of non-smoking LUSC patients (*p* = 0.033).

## Discussion

In our study, the connection between five variants in *CYP4F2* and LC risk in the Chinese Han population was detected. Association analyses revealed that *CYP4F2* rs12459936 increased susceptibility to LC in non-smoking individuals and females. In contrast, rs3093110 showed a protective effect on LC susceptibility in non-smoking groups and females. The two SNPs (rs3093193 and rs3093144) were also associated with a decreased risk of LC in females.

The *CYP4F2* gene, a member of the CYP450 superfamily, located on chromosome 19p13.12, has been shown to be expressed at higher levels in certain types of cancerous tissues, such as the thyroid, ovarian, breast, and colon (10). Eun et al. have confirmed that low expression of *CYP4F2* may contribute to the progression of hepatocellular carcinoma (HCC) and decrease survival rates due to its involvement in various metabolic pathways (15). A similar study showed that *CYP4F2* expression was higher in pancreatic ductal adenocarcinoma (PDA) patients than in normal ones and negatively correlated with age (16). Database prediction found that *CYP4F2* was highly expressed in lung cancer tissues. The expression of *CYP4F2* was higher in men than women and higher in non-smokers than smokers. Additionally, Xu et al. have reported that *CYP4F* generates 20-HETE by catalyzing ω-hydroxylation of arachidonic acid (17). According to previous studies, 20-HETE plays a significant role in tumor progression. Colombero et al. have demonstrated that HET0016, a selective inhibitor of 20-HETE synthesis, can reduce the proliferation of prostate cancer (18), while another study has revealed that the antagonist of 20-HETE, WIT002, is able to inhibit tumor growth in a renal cell carcinoma cell line (19). This suggests that *CYP4F2* polymorphisms may be related to susceptibility to LC by affecting the metabolism of 20-HETE, although further verification is required. Studies have also indicated a significant association between *CYP4F2* polymorphisms and a variety of diseases, including ischemic stroke and various other cardiovascular and cerebrovascular diseases (12, 20).

Our study focused on the association between *CYP4F2* polymorphisms and susceptibility to LC. Five sites were selected for statistical analyses: rs3093203, rs3093193, rs12459936, rs3093144, and rs3093110. However, none of these loci were found to be significantly associated with LC susceptibility under the allelic model or any of the five genetic models. The actual increase in LC risk may be underestimated due to the limited sample size. To further examine the potential influence of LC, we conducted a stratified analysis. Tobacco has long been recognized as an independent risk factor for tumorigenesis, as it contains many carcinogens, such as nitrosamines, polycyclic aromatic hydrocarbons, and volatile organic compounds (21). However, our analysis stratified by smoking revealed that the rs12459936 and rs3093110 loci were significantly associated with increased susceptibility to LC in the non-smoking population but not in the smoking population.

In addition, gender has been found to have a notable impact on the toxicity of therapeutic treatments and the response to them in many types of cancer. The underlying cause of this difference is likely related to a complex interplay of several factors, including sex hormones, which have been shown to affect the self-renewal of tumor stem cells, the tumor microenvironment, the immune system, and metabolism (22). It is well established that there are considerable differences in the immune system between men and women. In general, women have a stronger immune system than men, leading to distinct sex-based differences in both innate and adaptive immune responses. These disparities in immune systems likely play a role in cancer susceptibility between males and females (23). In our study, analysis stratified by gender was performed, and we found that rs309319, rs12459936, and rs3093110 all had a protective role against LC in females.

Taken together, our study observed that variants in *CYP4F2* were associated with LC susceptibility. However, our research had some limitations. First, the potential functional implications of *CYP4F2* polymorphisms were not addressed in this study. The expression data for *CYP4F2* in LC cases were sourced from the database. To properly elucidate the genetic mechanism of *CYP4F2* in LC, expression analysis of *CYP4F2* mRNA and annotation of the functional significance of variants are necessary. Second, the sample size was relatively small. In the following steps, we will perfect this information and expand the sample size to explore the molecular mechanism of *CYP4F2* polymorphisms affecting the development of LC.

## Data availability statement

The original contributions presented in the study are included in the article/**Supplementary Material**, further inquiries can be directed to the corresponding author/s.

## Ethics statement

Our study complied with the Declaration of Helsinki, and the protocol in our experience was approved by the Second Affiliated Hospital of Xi’an Jiaotong University. All participants have been informed and provided written informed consent for the study.

## Author contributions

HS designed this study and drafted the manuscript. YZ performed the DNA extraction and genotyping. YW revised the manuscript and performed the data analysis. PF and YL performed the samples collection and information recording. HS conceived and supervised the study. All authors contributed to the article and approved the submitted version.
