# Supplementary material for: Genetic polymorphisms in CYP4F2 may be associated with lung cancer risk among females and no-smoking Chinese population
Source: Front Oncol. 2023 Mar 14;13:1114218. doi: 10.3389/fonc.2023.1114218 (PMC10043406; doi:10.3389/fonc.2023.1114218)
Supplement: Supplementary file 1 [file Table_1.docx]

TableS1 Relationships of polymorphisms in *CYP4F2* genes and lung cancer susceptibility

| SNP ID | Model | Genotypes | Cases (%) | Controls (%) | OR (95% CI) | *p*-value |
| --- | --- | --- | --- | --- | --- | --- |
| rs3093203 | Genotype | GG | 286 (57.4%) | 295 (59.5%) | 1 |  |
|  |  | AA | 27 (5.4%) | 26 (5.2%) | 1.06 (0.60-1.87) | 0.836 |
|  |  | AG | 185 (37.1%) | 175 (35.3%) | 1.09 (0.83-1.41) | 0.542 |
|  | Dominant | GG | 286 (58.0%) | 295 (59.5%) | 1 |  |
|  |  | AA+AG | 212 (42.5%) | 201 (40.5%) | 1.08 (0.84-1.39) | 0.540 |
|  | Recessive | AG+GG | 471 (94.6%) | 470 (94.8%) | 1 |  |
|  |  | AA | 27 (5.4%) | 26 (5.2%) | 1.03 (0.59-1.79) | 0.923 |
|  | Additive | AA+AG+GG | - | - | 1.06 (0.86-1.31) | 0.586 |
| rs3093193 | Genotype | CC | 256 (50.5%) | 243 (48.2%) | 1 |  |
|  |  | GG | 41 (8.1%) | 42 (8.3%) | 0.94 (0.59-1.49) | 0.782 |
|  |  | GC | 210 (41.4%) | 219 (43.5%) | 0.91 (0.71-1.18) | 0.494 |
|  | Dominant | CC | 256 (50.5%) | 243 (48.2%) | 1 |  |
|  |  | GG+GC | 251 (49.5%) | 261 (51.8%) | 0.92 (0.72-1.18) | 0.494 |
|  | Recessive | GC+CC | 466 (91.9%) | 462 (91.7%) | 1 |  |
|  |  | GG | 41 (8.1%) | 42 (8.3%) | 0.98 (0.62-1.53) | 0.919 |
|  | Additive | GG+GC+CC | - | - | 0.94 (0.78-1.15) | 0.562 |
| rs12459936 | Genotype | CC | 148 (29.1%) | 155 (30.1%) | 1 |  |
|  |  | TT | 110 (21.7%) | 104 (20.6%) | 1.10 (0.78-1.57) | 0.582 |
|  |  | TC | 249 (49.1%) | 246 (48.7%) | 1.06 (0.80-1.41) | 0.679 |
|  | Dominant | CC | 148 (29.1%) | 155 (30.1%) | 1 |  |
|  |  | TT+TC | 359 (70.8%) | 350 (69.3%) | 1.07 (0.82-1.41) | 0.601 |
|  | Recessive | TC+CC | 397 (78.3%) | 401 (79.4%) | 1 |  |
|  |  | TT | 110 (21.7%) | 104 (20.6%) | 1.06 (0.79-1.44) | 0.692 |
|  | Additive | TT+TC+CC | - | - | 1.05 (0.88-1.25) | 0.572 |
| rs3093144 | Genotype | CC | 333 (65.7%) | 347 (68.7%) | 1 |  |
|  |  | TT | 16 (3.2%) | 16 (3.2%) | 1.05 (0.52-2.15) | 0.884 |
|  |  | TC | 158 (31.2%) | 142 (28.1%) | 1.16 (0.89-1.53) | 0.280 |
|  | Dominant | CC | 333 (65.7%) | 347 (68.7%) | 1 |  |
|  |  | TT+TC | 174 (34.3%) | 158 (31.3%) | 1.15 (0.88-1.50) | 0.295 |
|  | Recessive | TC+CC | 491 (96.9%) | 489 (96.8%) | 1 |  |
|  |  | TT | 16 (3.2%) | 16 (3.2%) | 1.01 (0.50-2.04) | 0.986 |
|  | Additive | TT+TC+CC | - | - | 1.11 (0.89-1.40) | 0.360 |
| rs3093110 | Genotype | AA | 408 (80.5%) | 380 (75.5%) | 1 |  |
|  |  | GG | 7 (1.4%) | 7 (1.4%) | 0.93 (0.32-2.69) | 0.896 |
|  |  | GA | 92 (18.1%) | 116 (23.0%) | 0.74 (0.55-1.01) | 0.058 |
|  | Dominant | AA | 408 (80.5%) | 380 (75.5%) | 1 |  |
|  |  | GG+GA | 99 (19.5%) | 123 (25.5%) | 0.75 (0.56-1.02) | 0.064 |
|  | Recessive | GA+AA | 500 (98.6%) | 496 (98.6%) | 1 |  |
|  |  | GG | 7 (1.4%) | 7 (1.4%) | 0.99 (0.35-2.86) | 0.992 |
|  | Additive | GG+GA+AA | - | - | 0.79 (0.60-1.04) | 0.092 |

Abbreviation: SNP: single-nucleotide polymorphisms; 95% CI: 95% confidence interval; OR: odds ratio

Notes: OR (95% CI) were calculated logistic regression analysis adjusted by gender and age.

.

Table S2 Associations of haplotype of *CYP4F2* and the risk of lung cancer

| SNP ID | Haplotypes | Controls-Fre | Cases-Fre | OR (95% CI) | *p*-value |
| --- | --- | --- | --- | --- | --- |
| rs3093203\|rs3093193\|rs12459936\|rs3093144\|rs3093110 | GCTCA | 0.445 | 0.457 | 1.000 | --- |
|  | ACCCA | 0.222 | 0.231 | 1.01 (0.80 - 1.26) | 0.950 |
|  | GGCTA | 0.167 | 0.180 | 1.05 (0.82 - 1.34) | 0.700 |
|  | GGCCG | 0.127 | 0.103 | 0.79 (0.59 - 1.06) | 0.120 |
|  | GCCCA | 0.026 | 0.015 | 0.57 (0.29 - 1.09) | 0.090 |

Abbreviation: SNP: single nucleotide polymorphism; Fre: Frequency OR: odds ratio; CI: confidence interval

OR (95% CI) were computed by logistic regression analysis adjusted by gender and age.
